# Avian movements in a modern world: cognitive challenges

**DOI:** 10.1007/s10071-016-1006-1

**Published:** 2016-06-10

**Authors:** Claudia Mettke-Hofmann

**Affiliations:** School of Natural Sciences and Psychology, Liverpool John Moores University, Liverpool, L3 3AF UK

**Keywords:** Environmental change, Cognition, Brain, Migrant, Nomad, Partial migrant

## Abstract

Different movement patterns have evolved as a response to predictable and unpredictable variation in the environment with migration being an adaptation to predictable environments, nomadism to unpredictable environments and partial migration to a mixture of predictable and unpredictable conditions. Along different movement patterns, different cognitive abilities have evolved which are reviewed and discussed in relation to an organism’s ability to respond to largely unpredictable environmental change due to climate and human-induced change, and linked to population trends. In brief, migrants have a combination of reliance on memory, low propensity to explore and high avoidance of environmental change that in combination with overall small brain sizes results in low flexibility to respond to unpredictable environmental change. In line with this, many migrants have negative population trends. In contrast, while nomads may use their memory to find suitable habitats, they can counteract negative effects of finding such habitats disturbed by large-scale exploratory movements and paying attention to environmental cues. They are also little avoidant of environmental change. Population trends are largely stable or increasing indicating their ability to cope with climate and human-induced change. Cognitive abilities in partial migrants are little investigated, but indicate attention to environmental cues coupled with high exploratory tendencies that allow them a flexible response to unpredictable environmental change. Indeed, their population trends are mainly stable or increasing. In conclusion, cognitive abilities have evolved in conjunction with different movement patterns and affect an organism’s ability to adapt to rapidly human-induced changes in the environment.

**Electronic supplementary material:**

The online version of this article (doi:10.1007/s10071-016-1006-1) contains supplementary material, which is available to authorized users.

## Introduction

Many bird species undertake large-scale movements to escape unfavourable conditions and/or utilise high-quality resources elsewhere. These movements can take the form of migration (to and fro migrations between breeding and wintering sites), partial migration when not all individuals in a population migrate and nomadism with birds following high-quality and abundant resources (Dean 2004). As a whole, large-scale movements are a global widespread phenomenon, and about 20 % of all bird species are migratory (Somveille et al. 2015). Three major Holarctic migration systems can be distinguished spanning all continents, the Nearctic–Neotropical system, the Palearctic–African system and the Palearctic–Asian system (Rappole and Jones 2003). Likewise, partial migration occurs worldwide (Jahn et al. 2012), but is particularly common in Australia with about 36 % of its bird species being partial migrants (Chan 2001). Nomadism is mainly linked to semi-arid and arid environments worldwide (Dean 2004) with nomadism accounting for about 10 % of all bird species in southern Africa (Dean 1997) and 26 % of all bird species in Australia (Smith 2015).

Climate change and human-induced activities result in rapidly changing environments. These changes are often unpredictable with extreme interannual variation in, for example, temperatures (e.g. Jalili et al. 2010). How bird species involved in large-scale movement fare in these ever-changing environments is of conservation importance. While many factors contribute to population decline including habitat loss, habitat change, fragmentation, pollution and others, a large part of a species’ reaction to environmental change is due to its ability to counteract these effects, that is, its ability to utilise new or different resources, adapt to other habitats or overcome gaps in its distribution. In this context, the paper will explore whether cognitive abilities play an important role in mitigating environmental change through flexible behavioural responses.

This article will review cognitive abilities of birds adopting different movement strategies and discuss them in relation to population developments and a species’ cognitive preparedness for an ever-changing world. I will first outline under which environmental conditions the different movement strategies evolved and then discuss cognitive adaptations and their consequences.

## Adaptation to variation in the environment

Migration has evolved as an adaptation to predictable seasonal environments (Dean 2004; Mettke-Hofmann 2014; Somveille et al. 2015) and often has an endogenous component determining the onset, direction and duration of migration (Gwinner 1986). Many migrants show morphological (e.g. longer wings), physiological (e.g. migratory fattening) and behavioural (e.g. nocturnal restlessness) adaptations to migration (Gwinner 1986; Leisler and Winkler 2003).

Partial migration describes populations where some individuals migrate and others do not (Lundberg 2013), and is an adaptation to seasonal environments with neither particularly harsh nor particularly benign winter conditions such as found at intermediate latitudes but with high variability (i.e. less predictability) in winter survival (Lundberg 1988). Whether an individual migrates can be genetically fixed or environmentally dependent and vary between years (Lundberg 1987). The proportion of birds migrating is often affected by environmental factors such as population density and resource availability with larger numbers migrating in years of high density and low resource availability (Nilsson et al. 2008).

Nomadism, in contrast, has evolved as an adaptation to unpredictable environmental conditions (Dean 1997). Nomads track superabundant but spatiotemporal unpredictable resources (Runge et al. 2015) and can often breed throughout the year whenever conditions are favourable (Jonzen et al. 2011).

Human-induced changes are from an animal’s perspective largely unpredictable in space and time, and climate change also results in increased uncertainty and variability in the environment (extreme weathers occur more often; Cormont et al. 2011). I, therefore, predict that nomadic species may be better equipped to withstand human-altered habitats as they are adapted to track unpredictable resources. Partial migrants also respond to environmental cues and may be able to buffer against environmental change, whereas migrants, particularly long-distance migrants, have evolved to cope with highly predictable (seasonal) changes and may be least adapted to unpredictable change.

## Cognitive abilities of birds with different movement strategies

This section will review what is known about cognitive adaptations to different movement strategies.

### Migratory species

Many migrants return to their breeding ground and often even the same territory year after year (e.g. Blums et al. 2002; Olalla-Kerstrupp et al. 2015). This requires a long-lasting memory for at least the period covering the non-breeding season. Indeed, long-distance migratory garden warblers (*Sylvia borin*) have been found to remember a room with food for at least 12 months, whereas closely related but resident Sardinian warblers (*Sylvia melanocephala*) remembered the same room for only 2 weeks (Mettke-Hofmann and Gwinner 2003). This long-lasting memory in migrants may even allow remembering high-quality stopover or wintering sites until the next year in accordance with winter site fidelity shown in many species (Rappole and Jones 2003; Paruk et al. 2015). Similarly, in dark-eyed juncos (*Junco hyemalis*) and white-crowned sparrows (*Zonotrichia leucophrys*), individuals of a migratory subspecies had a better spatial memory for previously visited food locations than individuals of a resident subspecies (Cristol et al. 2003; Pravosudov et al. 2006). The better spatial memory in the migrants was reflected in the hippocampal formation which is an important brain region for processing spatial information (Healy et al. 1994). The migrants among the dark-eyed juncos had more densely packed neurons in this region, and the migrants among the white-crowned sparrow had a larger hippocampal formation and also showed increased neurogenesis in this region than their resident counterparts (Cristol et al. 2003; Pravosudov et al. 2006; LaDage et al. 2011). Likewise, in the migratory garden warblers, relative hippocampal volume increased in migratory-experienced individuals as compared to migratory-naïve ones which indicates that the birds collected spatial information while on migration (Healy et al. 1996). The same study showed that hippocampal volume did not change in the resident Sardinian warbler. A recent study on two sandpiper species with different demands on visuospatial learning during migration also revealed a larger hippocampus and a larger number of microglia cells therein in the species with more demand on remembering visual cues during overland migration (*Actitis macularia*) as compared to *Calidris pusilla* with more non-stop flight over the Atlantic Ocean (Diniz et al. 2016). Overall, migrants seem to have a better spatial memory as an adaptation to their to and fro migration than residents.

While migrants have a larger hippocampus than residents, their overall brain size is smaller than in residents. Sol et al. (2010) found among 600 passerines that relative brain size decreased with migratory distance. Moreover, analyses indicated that migration selected for smaller brains, that is, migratoriness evolved from large-brained species, and then, selection favoured smaller brains (Sol et al. 2010). Similar results were found within species; in lark sparrows (*Chondestes grammacus*), migratory populations have smaller brains than resident populations (Fuchs et al. 2015). The reduced brain size may be an adaptation to reduce energy consumption (Winkler et al. 2004) as brains consume relatively more energy than other body parts (Laughlin et al. 1998). Alternatively, or in addition to this, the smaller brains are often linked to a flatter skull in migrants which may make the head more aerodynamic (Winkler et al. 2004).

The smaller brain size in migrants was also linked to less innovative behaviour in this group, particularly in long-distance migrants, as compared to short-distance migrants and residents (Sol et al. 2005). This indicates lower flexibility in behaviour in migrants as suggested by Sol (2003) and Winkler et al. (2004). Migrants are also less explorative than residents. Migratory garden warblers responded less to changes in their environment by taking longer to approach and investigate a novel object in their familiar environment than resident Sardinian warblers (Mettke-Hofmann et al. 2005a). They were, however, more likely to enter an unfamiliar environment, but once they were in this environment, they spent less time exploring it than the Sardinian warblers (Mettke-Hofmann et al. 2009). Moreover, the migrants covered more space per minute (Mettke-Hofmann and Gwinner 2004). These findings indicate that migrants are less hesitant to enter a novel environment which may be a prerequisite when encountering unfamiliar areas on migration, but that they do not invest a lot of time in exploring an unfamiliar environment. The exploration patterns reflect more superficial spatial exploration to get a rough overview about resources. These results are backed up by field studies showing that migrants move only over short distances in a straight line (i.e. covering unfamiliar territory rather than meandering around) and invest little in exploratory movements at stopover sites (Aborn and Moore 1997; Paxton et al. 2008). Migrants remain at each site for only relatively short periods of time (days to months depending whether it is a breeding, stopover or wintering site) and have to consider costs and benefits of exploration. While they have to collect information about suitable foraging sites and predation risk, they may keep this to a minimum as they cannot use this information in the long term (Mettke-Hofmann and Gwinner 2004; Mettke-Hofmann et al. 2012).

Several studies found that migrants use social information to get information about habitats quickly which may make up for the lower individual exploration. For example, migrants initially join flocks at stopover sites before foraging on their own (in insectivorous birds) which may serve information gathering by reducing uncertainty and risks associated with lack of information (Nemeth and Moore 2007). Likewise, some migrants use resident species as an indicator for high-quality breeding habitats and settlement decisions (heterospecific attraction hypothesis; Moenkkoenen et al. 1997). Moenkkoenen et al. (1999) suggested that this requires high cognitive abilities, for example, to recognise suitable species which has been shown in some migrants (Moenkkoenen et al. 1996).

While migrants show little spatial neophobia, that is, hesitancy to enter unfamiliar environments (Mettke-Hofmann et al. 2009), they are highly avoidant of changes in their familiar environment. A study comparing eight species/populations of sympatrically occurring New World blackbirds (Icteridae) during the non-breeding season showed strong avoidance reactions to feeding sites with novel objects placed around in migratory birds as compared to resident birds (Mettke-Hofmann et al. 2013). The same strong neophobia reaction was found in migratory garden warblers in comparison with resident Sardinian warblers (Mettke-Hofmann et al. 2005a). As migrants spend only limited time in each area, they may be more cautious about any changes as they do not know the risks/dangers associated with change, whereas residents may have a better knowledge whether, for example, a human-induced change is dangerous such as machinery placed in a field for future use.

Recent research has shown that individuals of a given species often differ consistently from each other in their response to environmental challenges (termed personality; for example, Koolhaas et al. 1999) which on the population level increases flexibility. Marchetti and Zehtindjiev (2009) suggested that in long-distance migratory sedge warblers (*Acrocephalus schoenobaenus*), personality can explain differences in behaviour during migration along a fear axis. While they did not test for within context repeatability, they found correlations across contexts that could best be explained with personality rather than other factors. Birds were captured during autumn migration and tested for their time to start foraging, migratory orientation and exploration of an unfamiliar room. Calmer birds (less escape movements) explored the cage more and were faster to accept food than birds moving more. Also, lean birds explored more. They suggested that the calm birds represent a reactive coping style with being more flexible and putting on less fat as they readily explore new environments. More nervous birds (more escape movements) represent a more proactive coping style with little flexibility but more reliance on fat reserves. Moreover, it has been shown in several bird species that individuals of the same species consistently differ in their timing of migration (Vardanis et al. 2016); that is, some individuals always migrate early, whereas others late. On the population level, this again provides flexibility to respond to environmental change. Interestingly, in black kites (*Milvus migrans*), timing of migration only became highly repeatable in adults, whereas juveniles progressively departed earlier each year (Sergio et al. 2014). Moreover, only early migrating individuals in their respective age class that were also able to advance their departure date in the first years survived and reproduced best, whereas individuals not able to advance their departure finally disappeared from the population. Unfortunately, it is unclear which factors (learning ability, body conditions or others) allowed earlier departures.

### Partially migratory species

Much less is known about cognitive abilities in partial migrants, but what is known contrasts in part with results just presented for obligate migrants. In partial migrants, not all individuals in a population migrate and whether to migrate individuals decided each year anew (Lundberg 2013). Partial migration should not be confused with species consisting of resident and migratory populations that have separate distributions where all individuals in each population express the same movement strategy. A study on partially migratory blue tits (*Cyanistes caeruleus*) showed that migrating individuals were more explorative of a novel object in their familiar environment than individuals that remained resident (Nilsson et al. 2010). Partial migrants respond much more strongly to environmental cues and may sample their environment for winter settlement suitability while on migration which may explain their higher exploration (Nilsson et al. 2010). There is also some evidence for a migratory versus resident personality in blue tits; individuals with a strong migratory propensity may initiate migration earlier and also start foraging in an unfamiliar environment earlier indicating that they settle in faster than individuals with a more resident personality, though no differences were found regarding neophobia (Nilsson et al. 2016). No other studies on personality are available for partially migratory birds; however, there are several studies in fish that indicate differences in personality between resident and migratory individuals. Migratory individuals in partially migratory fish were bolder (emergence from a hideout) and took more risk in an unfamiliar environment than resident individuals (Chapman et al. 2011). This again indicates that the migratory individuals among partial migrants are more open to novelty and invest in sampling unfamiliar environments than the resident individuals of the population. Nothing is known about other cognitive abilities in partially migratory birds. However, one may hypothesise that the migratory individuals have a better spatial memory than the resident individuals in the population to remember suitable wintering sites to the next season.

### Nomadic species

Finally, nomadic species show cognitive adaptations to their lifestyle. Movement patterns of some nomadic species indicate long-term memories for previously visited locations. For example, when current conditions deteriorated, grey teals (*Anas gracilis*) did not move to the next available wetland, but sometimes passed suitable sites to visit a remote wetland they may have visited earlier (Roshier et al. 2006). Likewise, snail kites (*Rostrhamus sociabilis*) are assumed to have a long-lasting memory for wetlands (Bennetts and Kitchens 2000). In contrast, memory was not considered to be important in two seedeater species (*Sporophila*) as seeding cycles of bamboo plants (the main food of the species) were longer than the average lifespan of the birds. While these plants have predictable cycle intervals, plants are not synchronised making the spatiotemporal occurrence of seeding unpredictable and with the very long cycle intervals, often around 20 years, a bird’s life is too short to learn about the cycle length of particular plants (Areta et al. 2013).

Snail kites were shown to have higher movement patterns during good food conditions which were interpreted as exploration movements and may serve to increase knowledge about the environment as lakes dry out every 5–10 years (Bennetts and Kitchens 2000). Conducting such flights during good periods reduces the costs of exploration. In contrast, on a local scale (exploration of a neighbouring aviary), nomadic parrot species showed less spatial exploration and also explored changes in their familiar environment less than closely related resident species (Mettke-Hofmann et al. 2005b, 2012). Like in migratory species, short residency times may make extensive local exploration and changes therein too costly. However, nomadic species seem to have similarly sized brains as residents. Based on data of movement patterns in parrots (Mettke-Hofmann et al. 2002), brain sizes (Iwaniuk et al. 2005) were compared between pairs of closely related species (one a nomad, the other a resident; when more than one species of a particular movement type was available within a group, the mean was used) resulting in nine pairings. Results indicate that residents and nomads have similar brain sizes (paired *t* test: *n* = 9, d*f* = 8, *t* = 0.927, *p* = 0.381; Table 1). This may give nomadic species the necessary flexibility to respond to unpredictable environmental change.

**Table 1:** Relative brain size of resident and nomadic parrot species

Species	Movement pattern	Pairs	Brain/body ratio (ml/g)	Brain/body ratio (ml/g)	
				Residents	Nomads
*Cacatua alba*	R	1	0.0224405705	0.0224405705	0.0184667300
*Cacatua g. galerita*	N	1	0.0186143791		
*Cacatua roseicapilla*	N	1	0.0183190883		
*Psephotus haematonotus*	R	2	0.0332770270	0.0332770270	0.0315696649
*Psephotus varius*	N	2	0.0315696649		
*Barnardius b. barnardi*	R	3	0.0222632226	0.0222632226	0.0294813467
*Platycercus flaveolus*	N	3	0.0294813467		
*Neopsittacus musschenbroekii*	R	4	0.0429824561	0.0337134503	0.0335563984
*Lorius garrulus*	R	4	0.0244444444		
*Trichoglossus ornatus*	N	4	0.0292500000		
*Glossopsitta concinna*	N	4	0.0378627968		
*Chalcopsitta cardinalis*	N	5	0.0262500000	0.0396666667	0.0259690367
*Pseudeos fuscata*	N	5	0.0256880734		
*Eos squamata*	R	5	0.0383000000		
*Eos bornea*	R	5	0.0398333333		
*Psittacula columboides*	R	6	0.0381111111	0.0381111111	0.0269230769
*Psittacula alexandri*	N	6	0.0269230769		
*Agapornis taranta*	R	7	0.0344347826	0.0384729357	0.0368007663
*Agapornis roseicollis*	R	7	0.0406113537		
*Agapornis fischeri*	R	7	0.0403726708		
*Agapornis lilianae*	N	7	0.0377777778		
*Agapornis personata*	N	7	0.0358237548		
*Bolborhynchus lineola*	N	8	0.0388059701	0.0336956522	0.0388059701
*Poicephalus cryptoxanthus*	R	8	0.0336956522		
*Amazona vinacea*	R	9	0.0181199903	0.0192923669	0.0203504380
*Amazona autumnalis*	N	9	0.0203504380		
*Amazona o. auropalliata*	R	9	0.0221016166		
*Amazona farinosa*	R	9	0.0166229508		
*Amazona leucocephala*	R	9	0.0203249097		
Overall mean (± SE)				0.031214778 ± 0.00194	0.029102603 ± 0.00171

Movement pattern: R = resident; N = nomadic; arrangement into closely related pairs of residents and nomads followed the phylogeny in Mettke-Hofmann et al. (2002); brain/body ratios are from Ivaniuk et al. (2005)

Unlike migrants, nomadic species seem to be less avoidant of changes in their familiar environment. Data on neophobic reactions of over fifty parrot species (Mettke-Hofmann et al. 2002) were reanalysed by planned comparisons of closely related species by excluding phylogenetic groups consisting of purely resident or nomadic species. Additionally, a paired comparison (one a nomad, the other a resident; see above) was conducted which resulted in 11 pairings. This way, taxon-specific effects in neophobia reactions were avoided though phylogeny did not play a role in the full data set (Mettke-Hofmann et al. 2002). While overall residents and nomads did not differ systematically in their neophobic reaction (latency to forage beside a novel object; *t* test: *n* = 29, df = 27, *t* = 1.213, *p* = 0.236), in all pairings, nomads were less neophobic than closely related resident parrot species (paired *t* test: *n* = 11, df = 10, *t* = −3.132, *p* = 0.011). This is an interesting finding as it indicates that nomads are not afraid of changes in their environment which is in strong contrast with neophobic reactions in migrants.

Nomadic species seem to pay attention to a variety of environmental cues to decide about where to go when conditions deteriorate, whereas migratory species generally follow endogenous programmes (Gwinner 1986) though partial migrants also use local cues to decide about when to migrate (Nilsson et al. 2006). At least for nomadic wetland birds, it has been suggested that they use visual cues such a cloud formation, or changes in temperature and pressure gradients that predict rainfall (Simmons et al. 1998) for decision-making whether and where to move. Roshier et al. (2006) suggested that primary productivity of algae and other micro-organisms would lead to a distinctive olfactory signal which could be picked up by nomadic species over large distances. There are no personality studies available in relation to movements in nomads.

## Cognitive adaptations in a modern world

This section will discuss how birds with cognitive adaptations to different migration strategies fare in our rapidly changing environment where changes are often unpredictable.

### Migrants

**Fig. 1:**
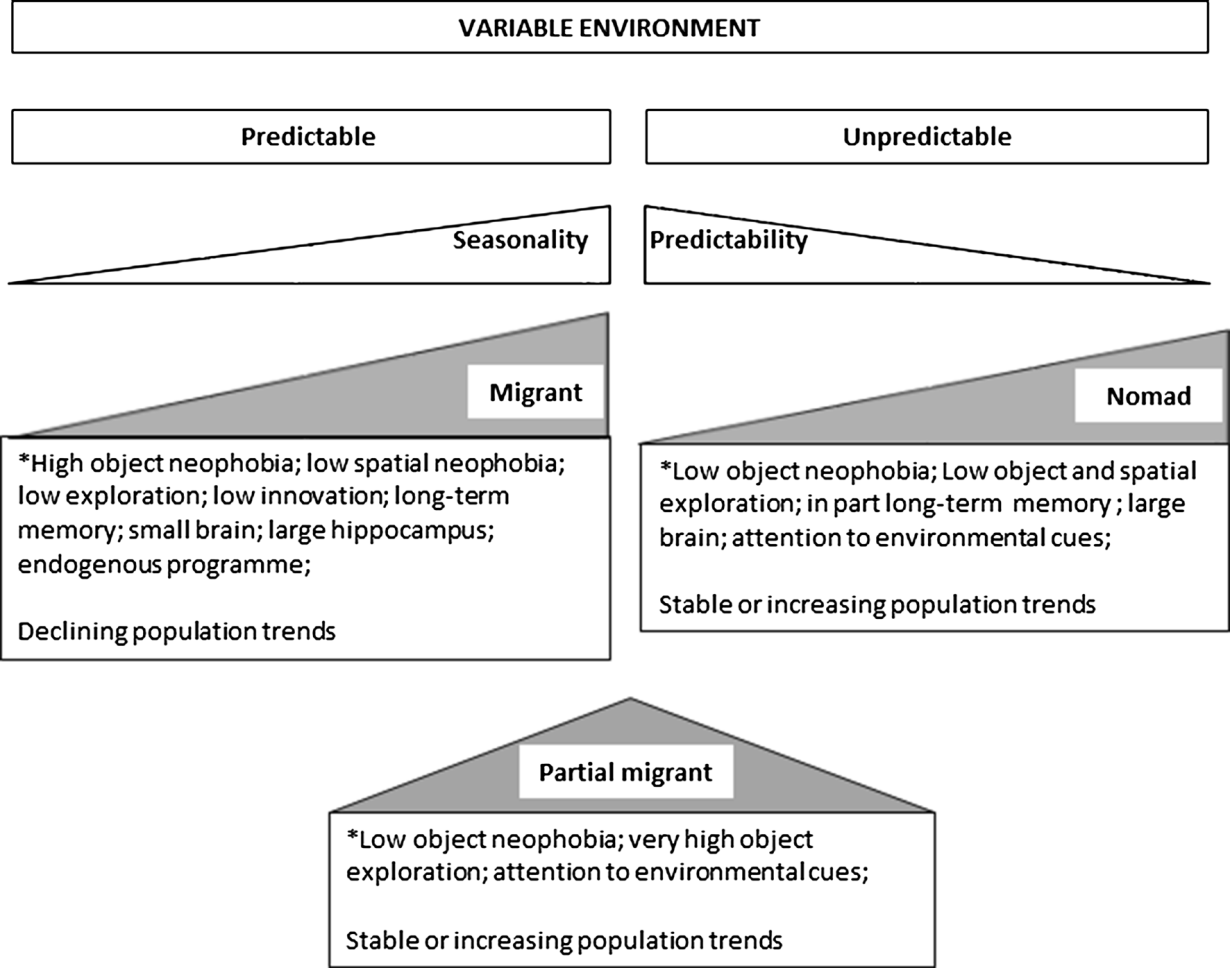
Cognitive abilities, movement patterns and environmental variation. Environmental variation can be predictable or unpredictable. Migrants have evolved as an adaptation to highly seasonal environments, whereas nomads are adapted to unpredictable environments. Partial migrants evolved in seasonal environments with a high degree of stochasticity. Different movement patterns correlate with specific cognitive abilities. *Asterisk* Cognitive abilities are described in comparison with closely related resident species

Migrants have evolved cognitive adaptations to deal with predictable variation in the environment (Fig. 1). This includes a long-lasting memory for high-quality stopover sites, last year breeding territories and former wintering sites. While this is advantageous in a predictable environment, it can be a disadvantage when environments become less predictable due to climate change or human activity (Cormont et al. 2011). For example, stopover and breeding sites may have disappeared, shifted or changed and site fidelity can be, in the best case, a waste of time (as birds return to a site that is less suitable now) or, in the worst case, mean death when habitats may have become unsuitable (Cresswell 2014). The lower exploration propensity in combination with their smaller overall brains also makes migrants less likely to find suitable habitats or adapt to changed habitats. Indeed, migrants largely settle along their migration pathway and underuse suitable habitat further away from this path (Telleria et al. 2008). However, some plasticity has been reported in pink-footed geese (*Anser brachyrhynchus*) where birds moved on to nearby staging sites after arriving at a former autumn and winter site that had disappeared due to restoration of a lake (Clausen and Madsen 2016). Nonetheless, migrants as a group were found to have the lowest invasion success when introduced to New Zealand (Sol and Lefebvre 2000). Their strong neophobia to changes in the environment keeps them away from possible resources and may restrict them to more pristine areas. While all these cognitive abilities make it difficult for a migrant to respond to environmental change, their ability to use other species as a cue for good habitat conditions (Moenkkoenen et al. 1997; Nemeth and Moore 2007) may buffer these disadvantages to some extent. Moreover, at least some migrants vary in behaviour along personality axes with some individuals being more explorative (Marchetti and Zehtindjiev 2009) which may allow for rapid selection. However, this does not seem enough to counter negative population trends.

Among the European breeding birds (*n* = 350), population trends of long-distance migrants were significantly more negative than of short-distance migrants and residents, irrespective of breeding or wintering habitat or continent (Sanderson et al. 2006). Similarly, a study by Lloyd-Evans and Atwood (2004) on 78 migratory species in America showed a negative population trend in the majority of the migratory species. Moreover, Holmes and Sherry (2001) showed that particularly long-distance migrants in America (*n* = 24 species) are declining. Long-distance migrants may be at a particular disadvantage as cues used to initiate spring migration may be misaligned with conditions on the breeding ground as a consequence of climate change (van Turnhout et al. 2010; Cormont et al. 2011), and migration may be overall more endogenously controlled than in short-distance migrants that have the ability to respond to environmental cues more flexibly (Sol et al. 2005). The same pattern of the highest proportion of declining populations in migrants as compared to all other groups was also found in the bird taxa listed in the Online Resources 1.

While migrants span a large range which may increase the risk of encountering habitat change and loss, and may affect population development of migrants more than residents, cognitively they seem to be poorly adapted to respond to those challenges. The combination of low exploration, reliance on memory rather than exploration and strong avoidance reactions to changes makes this group particularly vulnerable to unpredictable environmental change as they are behaviourally less flexible and may have a low propensity of bold individuals.

### Partial migrants

The little that is known about cognitive abilities in partial migrants seems to prepare them for unpredictable environments (Fig. 1) which contrasts strongly with what is known from obligate migrants. The high exploration propensity and attention paid to environmental conditions in both, resident and migratory individuals among partial migrants, makes them well adapted to unpredictable environmental change as they can react flexibly to current situations (Chan 2001). Actually, partial migration is assumed to have evolved as a response to though seasonal but unpredictable variation in, for example, population density and food availability across years (Lundberg 1988). Moreover, models predict partial migration to evolve under strong environmental stochasticity (Velez-Espino et al. 2013). The authors conclude that partial migration can serve as a buffer against environmental stochasticity. This seems to be supported by population developments in partial migrants as they remain largely stable or even increase (Online Resource 1) indicating that they cope relatively well with human-altered habitats. An example is partially migratory blue tit populations in Sweden which have increased over the last 30 years (Nilsson et al. 2006). Interestingly, the proportion of migratory blue tits has also increased in contrast to predictions that global warming should reduce the migratory proportion (Berthold 2003) indicating density-dependence in this system (Velez-Espino et al. 2013). Moreover, their ability to cope with unfamiliar environments is further supported by their high invasion success (Sol and Lefebvre 2000). The possible existence of migratory and resident personality types may help during this process as different coping styles are suggested to be important in mastering different stages of invasion (Chapple et al. 2012). At least among partially migratory blue tits, some of the migratory individuals (17 %) do not return to their breeding ground (Nilsson et al. 2008). Their high exploration propensity and ability to flexibly decide whether or not to migrate allow them settling in new areas. Overall, partial migrants seem to be cognitively much better prepared to respond to human-altered habitats than obligate migrants. This seems to be the case for both, resident and migratory individuals, among partially migratory populations which is in stark contrast to species consisting of resident and migratory populations that are either adapted to residency or migratoriness.

### Nomads

Nomadic species have evolved cognitive abilities to track superabundant but highly unpredictable resources (Fig. 1). As such, they seem to be predisposed to fare well in human-altered habitats. But is this really the case? The long-term spatial memory may help nomads to decide where to go when local conditions deteriorate. However, like in the migrants they may head to an area which is not suitable any more (Cresswell 2014). Nonetheless, they may be better off than migrants as they respond strongly to environmental cues (even over large distances; for example, Roshier et al. 2006), show large-scale exploratory movements (Bennetts and Kitchens 2000), have similarly sized brains as residents and often move in groups (Dean 1997) which may increase their chances of finding suitable sites. While nomads are often diet and habitat specialists (Dean 1997) and, therefore, are less resilient to environmental change than residents (Runge et al. 2015), their ability to dynamically respond to variable environmental conditions by large-scale movements may counteract this specialisation. Indeed, nomadic Worthen’s sparrows (*Spizella wortheni*) which occur in semi-arid and arid areas in Mexico maintained high genetic diversity with nearly no differentiation between sites despite considerable habitat fragmentation due to their large-scale movements (Canales-Delgadillo et al. 2012). Simmons et al. (2004) speculate that nomadic species may counteract climate change by moving away from unsuitable habitats.

Nomads also seem to be little afraid of changes in their environment, even less than residents in a direct comparison which is in stark contrast to reactions in migrants. As a consequence, they may be less stressed by human-induced changes and may tolerate human activity (e.g. trumpeter hornbills (*Bycanistes bucinator*) move into agricultural landscapes during the non-breeding season; Lenz et al. 2015). Their low neophobia may help them to settle relatively easy in captive environments where nomadic species such as the zebra finch (*Taeniopygia guttata*), the cockatiel (*Nymphicus hollandicus*) or the budgerigar (*Melopsittacus undulatus*) are among the most successful and easy to keep bird species. Nomadic species as a group also seem to cope relatively well with environmental change. Dean (2004) stated that none of the nomadic species are classified as threatened or endangered which is confirmed in the selection in the Online Resource 1, though four species were classed as either near threatened or vulnerable. Moreover, their population development is largely stable or even increasing (Online Resource 1). Overall, the ability of nomads to respond to environmental cues, decide about movement decisions flexibly and collect large-scale information about the environment during extended movements seems to prepare them well for climate change-induced environmental challenges and human-induced changes such as habitat fragmentation.

## Conclusion

Different movement patterns have evolved as a response to predictable and unpredictable variation in the environment accompanied by specific cognitive abilities to deal with the adversaries of a mobile life. It seems that it is not movement *per se* that disadvantage an organism in a rapidly changing environment, but it is the adaptation to specific environmental conditions (e.g. predictable variable environments) resulting in a particular combination of cognitive traits that may leave some groups behind. For instance, the combination of relying on memory in combination with little propensity to explore and high avoidance of changes in the familiar environment leaves migrants with only little room to respond to unpredictable environmental change. In contrast, disadvantages of spatial memory in nomads can be counteracted by flexibility where to go and attention paid to environmental stimuli. Their low neophobia also makes them less susceptible to environmental change. While species from all movements patterns (including residents) suffer from habitat loss, species with cognitive adaptations to unpredictable environmental conditions such as partial migrants and nomads seem to be able to respond more flexibly to climate and human-induced change (e.g. habitat fragmentation) than migrants that have adapted to predictable conditions, thus confirming the initial hypothesis. To buffer the negative impact of environmental change particularly on migrants, conservation efforts require maintaining suitable, non-disturbed (or only slightly disturbed) habitats on the breeding and overwintering ground and when on migration.

## Electronic supplementary material

Below is the link to the electronic supplementary material.

Supplementary material 1 (PDF 236 kb)
