# Supplementary material for: Avian movements in a modern world: cognitive challenges
Source: Anim Cogn. 2016 Jun 10;20(1):77–86. doi: 10.1007/s10071-016-1006-1 (PMC5274642; doi:10.1007/s10071-016-1006-1)
Supplement: Supplementary file 1 — Supplementary material 1 (PDF 236 kb) [file 10071_2016_1006_MOESM1_ESM.pdf]

Article title: Avian movements in a modern world – cognitive challenges

Journal name: Animal Cognition

Author: Claudia Mettke-Hofmann

Affiliation: School of Natural Sciences & Psychology, Liverpool John Moores University, United Kingdom

Email: c.c.mettke-hofmann@ljmu.ac.uk

Online Resource 1: Assemblage of bird species with different movement patters and their population development and conservation status

The table assembles closely related species (to reduce phylogenetic effects) that differ in their movement patters. It allows comparing population trends of species with different movement patterns (published literature only covers population trends of migrants) by applying the same criteria to all species. Selection of species was initially focussed on nomadic and partially migratory species (Dean 1997; Bennets and Kitchen 2000; Roshier et al. 2006; Pavey and Nano 2009; Cormont et al. 2011; Canales-Delgadillo et al. 2012; Areta et al. 2013) as they were assumed to be more difficult to find and little published information about population trends in these groups was available. Their movement pattern was then validated with del Hoyo et al. (1992 - 2002a; vol 1-7) and del Hoyo et al. (2003 – 2011b; vol 8-16). Then, closely related species with resident and migratory movement pattern were added using del Hoyo et al. (1992 - 2002a; vol 1-7) and del Hoyo et al. (2003 – 2011b; vol 8-16). Finally, additional taxonomic groups were added using the same sources as mentioned before to have a broad range of taxons included. Nonetheless, it is acknowledged that the table represents only a small proportion of species with different movement patterns.

**Species**	**Res**	**Par**	**Migr**	**Nom**	**Status**	**Region**
Struthioniformes						
Struthionidae						
Ostrich *Struthio camelus*				D	LC	Afr
Rheidae						
Greater rhea *Rhea americana*	D				NT	SAm
Casuariidae						
Dwarf cassowary *Casuarius bennetti*	S				LC	As
Dromaiidae						
Emu *Dromaius novaehollandiae*				S	LC	Au
Podicipediformes						
Podicipedidae						
Red-necked grebe *Podiceps grisegena*			D		LC	Eu
Junin flightless grebe *Podiceps taczanowskii*	D				CE	SAm
Little grebe *Tachybaptus ruficollis*		D			LC	Eu
Australasian grebe *Tachybaptus novaehollandiae*				I	LC	Au
Pelecaniformes						
Phalacrocoracidae						

**Species**	**Res**	**Par**	**Migr**	**Nom**	**Status**	**Region**
Great cormorant *Phalacrocorax carbo*		I			LC	Eu
Double-crested cormorant *Phalacrocorax auritus*			I		LC	Am
Neotropical cormorant *Phalacrocorax olivaceus*	I				LC	SAm
Guanay cormorant *Phalacrocorax bougainvillii*				D	NT	SAm
Ciconiiformes						
Ardeidae						
White-necked Heron *Ardea pacifica*				S	LC	Au
Little egret *Egretta garzetta*		I			LC	As
Tricolored heron *Egretta tricolor*			S		LC	Am
Eastern reef egret *Egretta sacra*	S				LC	As
Anseriformes						
Anatidae						
Australian wood duck *Chenonetta jubata*				S	LC	Au
Grey teal *Anas gracilis*				D	LC	Au
African black duck *Anas sparsa*	D				LC	Afr
Northern shoveler *Anas clypeata*			D		LC	Eu
Mallard *Anas platyrhynchos*		D			LC	Eu
Red shoveler *Anas platalea*		S			LC	SAm
Yellow-billed duck *Anas undulata*	S				LC	Afr
Northern pintail *Anas acuta*			D		LC	Eu
Tufted duck *Aythya fuligola*		S			LC	Eu
Ferruginous duck *Aythya nyroca*			D		NT	Eu
Hardhead *Aythya australis*				S	LC	Au
New Zealand scaup *Aythya novaeseelandiae*	I				LC	NZ
Madagascar pochard *Aythya innotata*	S				CE	Afr
Mute swan *Cygnus olor*		I			LC	Eu
Whooper swan *Cygnus cygnus*			I*		LC	Eu
Trumpeter swan *Cygnus buccinator*	I				LC	Am
Falconiformes						
Accipitridae						
Square-tailed Kite *Lophoictinia isura*			S		LC	Au
Common Black-shouldered Kite *Elanus caeruleus*				S	LC	Afr
Whistling Kite *Haliastur sphenurus*		D			LC	Au
Brahminy kite *Haliastur indus*	D				LC	Au
Spotted Harrier *Circus assimilis*				S	LC	Au
African marsh-harrier *Circus ranivorus*	D				LC	Afr
Hen harrier *Circus cyaneus*			D		LC	Eu
Brown Goshawk *Accipiter fasciatus*		D			LC	Au
Crested goshawk *Accipiter trivirgatus*	D				LC	As
Collared Sparrowhawk *Accipiter cirrocephalus*	D				LC	Au
Chinese goshawk *Accipiter soloensis*			D		LC	As
Sparrowhawk *Accipiter nisus*		S			LC	Eu
Levant sparrowhawk *Accipiter brevipes*			S		LC	Eu
Buzzard *Buteo buteo*		S			LC	Eu
Rough-legged buzzard *Buteo lagopus*			S		LC	Eu
Augur buzzard *Buteo augur*	S				LC	Afr
Wedge-tailed Eagle Aquila audax	I				LC	Au
Tawny eagle *Aquila rapax*				D	LC	Afr

Species	Res	Par	Migr	Nom	Status	Region
Wahlberg's eagle *Aquila wahlbergi*			S		LC	Afr
Verreaux 's eagle *Aquila verreauxii*	S				LC	Afr
Lesser spotted eagle *Aquila pomarina*			S		LC	Eu
Falconidae						
Grey falcon *Falco hypoleucos*		S			V	Au
Brown falcon *Falco berigora*	D				LC	Au
Hobby *Falco subbuteo*			D		LC	Eu
Kestrell *Falco tinnunculus*		D			LC	Eu
Greater kestrel *Falco rupicoloides*				S	LC	Afr
Grey kestrel *Falco ardosiaceus*	S				LC	Afr
Sooty falcon *Falco concolor*			D		NT	Afr
Galliformes						
Phasianidae						
Stubble quail *Coturnix pectoralis*				I	LC	Au
Japanese quail *Coturnix japonica*			D		NT	As
Gruiformes						
Turnicidae						
Little Button-quail *Turnix velox*				D	LC	Au
Black-breasted buttonquail *Turnix melanogaster*	D				NT	Au
Rallidae						
Water rail *Rallus aquaticus*		D			LC	Eu
Clapper rail *Rallus longirostris*	D	D			LC	Am
King rail *Rallus elegans*			D		NT	Am
Plain-flanked rail *Rallus wetmorei*	D				E	SAm
Coot *Fulica atra*			D		LC	Eu
Red-knobbed coot *Fulica cristata*				D	LC	Eu
American coot *Fulica americana*			D		LC	Am
Red-fronted coot *Fulica rufifrons*	S				LC	SAm
Giant coot *Fulica gigantea*	S				LC	SAm
Charadriiformes						
Charadriidae						
Chestnut-banded plover *Charadrius pallidus*				S	NT	Afr
Wilson's plover *Charadrius wilsonia*		D			LC	SAm
Collared plover *Charadrius collaris*	D				LC	SAm
Common ringed plover *Charadrius hiaticula*			D		LC	Eu
Shore plover *Thinornis novaeseelandiae*	I				E	NZ
Pteroclidiformes						
Pteroclididae						
Namaqua sandgrouse *Pterocles namaqua*				S	LC	Afr
Burchell's sandgrouse *Pterocles burchelli*				S	LC	Afr
Double-banded sandgrouse *Pterocles bicinctus*	D				LC	Afr
Yellow-throated sandgrouse *Pterocles gutturalis*		D			LC	Afr
Pallas's sandgrouse *Syrrhaptes paradoxus*		S			LC	As
Tibetan sandgrouse *Syrrhaptes tibetanus*	D				LC	As
Columbiformes						
Columbidae						
Common bronzewing *Phaps chalcoptera*				S	LC	Au
Brush bronzewing *Phaps elegans*	D				LC	Au

Species	Res	Par	Migr	Nom	Status	Region
Flock bronzewing *Phaps histrionica*				D	LC	Au
Crested Pigeon *Ocyphaps lophotes*	I				LC	Au
Spinifex Pigeon *Geophaps plumifera*	S				LC	Au
Patridge pigeon *Geophaps smithii*				D	V	Au
Diamond Dove *Geopelia cuneata*				S	LC	Au
Bar-shouldered dove *Geopelia humeralis*	S				LC	Au
Stock pigeon *Columba oenas*		I			LC	Eu
Wood pigeon *Columba palumbus*		I			LC	Eu
Rock dove *Columba livia*	D				LC	Eu
African Olive-pigeon *Columba arquatrix*				D	LC	Afr
Hill pigeon *Columba rupestris*	D				LC	As
Ashy woodpigeon *Columba pulchricollis*				D	LC	As
Spot-winged pigeon *Columba maculosa*			I		LC	SAm
Picazuro pigeon *Columba picazuro*			I		LC	SAm
Psittaciformes						
Cacatuidae						
Red-tailed Black-Cockatoo *Calyptorhynchus*				D	LC	Au
Yellow-tailed black cockatoo *Calyptorhynchus*				S	LC	Au
Gang-gang *Callocephalon fimbriatum*			I		LC	Au
Major Mitchell's Cockatoo *Cacatua leadbeateri*				S	LC	Au
Galah *Eolophus roseicapillus*				I	LC	Au
Little Corella *Cacatua sanguinea*				I	LC	Au
Long-billed corella *Cacatua tenuirostris*	I				LC	Au
Sulphur-crested cockatoo *Cacatua galerita*	D				LC	Au
Cockatiel *Nymphicus hollandicus*				S	LC	Au
Psittacidae						
Australian Ringneck *Barnardius zonarius*				I	LC	Au
Red-capped parrot *Purpureicephalus spurius*	I				LC	Au
Mulga Parrot *Psephotus varius*				D	LC	Au
Red-rumped parrot *Psephotus haematonotus*	I				LC	Au
Bourke's Parrot *Neopsephotus bourkii*				I	LC	Au
Blue-winged parrot *Neophema chrysostoma*			S		LC	Au
Rock parrot *Neophema petrophila*	D				LC	Au
Budgerigar *Melopsittacus undulatus*				I	LC	Au
Red-masked parakeet *Aratinga erythrogenys*			D		NT	SAm
Orange-fronted parekeet *Aratinga canicularis*				S	LC	SAm
Socorro parakeet *Aratinga brevipes*	D				LC	SAm
Strigiformes						
Strigidae						
Short-eared owl *Asio flammeus*				D	LC	Eu
Striped owl *Asio clamator*	S				LC	SAm
Marsh owl *Asio capensis*		S			LC	Afr
Caprimulgiformes						
Caprimulgidae						
Great Eared Nightjar *Eurostopodus macrotis*	S				LC	As
White-throated nightjar *Eurostopodus mystacalis*		D			LC	Au
Chuck-will's widow *Caprimulgus carolinensis*			D		LC	Am
Scrub nightjar *Caprimulgus anthonyi*				I	LC	SAm

Species	Res	Par	Migr	Nom	Status	Region
Passeriformes						
Alaudidae						
red-capped lark, *Calandrella cinerea*				I	LC	Afr
Greater short-toe lark *Calandrella brachydactyla*			D		LC	Eu
Hume’s lark *Calandrella acutirostris*			S		LC	As
Sand lark *Calandrella raytal*	S				LC	As
Lesser Short-toes lark *Calandrella rufescens*		D			LC	Eu
Dunn’s lark *Eremalauda dunni*	S				LC	Afr
Pink-billed lark *Spizocorys conirostris*				D	LC	Afr
Stark's lark *Spizocorys starki*				S	LC	Afr
Botha’s lark *Spizocorys fringillaris*	D				E	Afr
Chestnut-backed sparrow-lark *Eremopterix*				S	LC	Afr
grey-backed sparrow-lark *Eremopterix verticalis*				S	LC	Afr
Black-eared Sparrow Lark *Eremopterix australis*				D	LC	Afr
Ashy-crowned sparrowlark *Eremopterix griseus*	S				LC	As
Chestnut-headed sparrowlark *Eremopterix*	I				LC	Afr
Singing bushlark *Mirafra javanica*	S				LC	Au
Jerdon’s bushlark *Mirafra affinis*	S				LC	As
Singing bushlark *Mirafra cantillans*		D			LC	Afr
Kordofan lark *Mirafra cordofanica*				D	LC	Afr
Campephagidae						
Ground Cuckoo-shrike *Coracina maxima*				D	LC	Au
Black-faced Cuckoo-shrike *Coracina*			D		LC	Au
Boyer’s Cuckoo-shrike *Coracina boyeri*	S				LC	As
Black-headed Cuckoo-shrike *Coracina*		S			LC	As
White-winged Triller *Lalage sueurii*				I	LC	Au
White-winged triller *Lalage tricolor*		S			LC	Au
Varied triller *Lalage leucomela*	D				LC	Au
Turdidae						
Blackbird *Turdus merula*		S			LC	Eu
Song thrush *Turdus philomelos*		I			LC	Eu
Mistle thrush *Turdus viscivorus*		D			LC	Eu
Redwing *Turdus iliacus*			D		NT	Eu
Island thrush *Turdus poliocephalus*	D				LC	As
Creamy-bellied thrush *Turdus amaurochalinus*		S			LC	SAm
Black-billed thrush *Turdus ignobilis*	S				LC	SAm
Black-billed thrush *Turdus ignobilis*	S				LC	SAm
Fieldfare *Turdus pilaris*				S	LC	Eu
Ring ouzel *Turdus torquatus*			D		LC	Eu
Grey-sided thrush *Turdus feae*			D		V	As
Chinese thrush *Turdus mupinensis*	S				LC	As
Plumbeous-backed thrush *Turdus reevei*				D	LC	SAm
Muscicapidae						
Pied flycatcher *Ficedula hypoleuca*			D		LC	Eu
Rufous-chested flycatcher *Ficedula dumetoria*	D				LC	As
Spotted flycatcher *Muscicapa striata*			D		LC	Eu
Gambaga flycatcher *Muscicapa gambagae*		S			LC	Afr
Sumba brown flycatcher *Muscicapa segregata*	D				NT	As

**Species**	**Res**	**Par**	**Migr**	**Nom**	**Status**	**Region**
Regulidae						
Fire crest *Regulus ignicapillus*		S			LC	Eu
Gold crest *Regulus regulus*			D		LC	Eu
Madeira firecrest *Regulus madeirensis*	D				LC	Eu
Sylviidae						
Rufous Songlark *Cincloramphus mathewsi*			S		LC	Au
Brown Songlark *Cincloramphus cruralis*				S	LC	Au
Spinifexbird *Eremiornis carteri*	D				LC	Au
Marsh grassbird *Megalurus pryeri*		D			NT	As
Paridae						
White-winged black tit *Parus leucomelas*	S				LC	Afr
Blue tit *Cyanistes caeruleus*		I			LC	Eu
Ashy tit *Parus cinerascens*				S	LC	Afr
Remizidae						
Penduline tit *Remiz pendulinus*		I			LC	Eu
White-crowned penduline tit *Remiz coronatus*			D		LC	As
Black-headed penduline tit *Remiz macronyx*	S				LC	As
Meliphagidae						
Pied Honeyeater *Certhionyx variegatus*				S	LC	Au
Varied honeyeater *Lichenostomus versicolor*	S				LC	Au
White-plumed Honeyeater *Lichenostomus*	D				LC	Au
Yellow-faced honeyeater *Lichenostomus*		D			LC	Au
White-fronted Honeyeater *Purnella albifrons*				S	LC	Au
Yellow-throated Miner *Manorina flavigula*	S				LC	Au
Spiny-cheeked Honeyeater *Acanthagenys*	D				LC	Au
Regent honeyeater *Anthochaera phrygia*			D		CE	Au
Rufous-throated honeyeater *Conopophila*				S	LC	Au
Black honeyeater *Sugomel nigrum*			D		LC	Au
Scalet myzomela *Myzomela sanguinolenta*		S			LC	Au
Banded honeyeater *Cissomela pectoralis*				S	LC	Au
White-naped honeyeater *Melithreptus lunatus*		S			LC	Au
Painted honeyeater *Grantiella picta*			D		V	Au
Artamidae						
Masked Woodswallow *Artamus personatus*				S	LC	Au
Black-faced Woodswallow *Artamus cinereus*	I				LC	Au
Little Woodswallow *Artamus minor*		S			LC	Au
Dusky woodswallow *Artamus cyanopterus*			D		LC	Au
Corvidae						
Little Crow *Corvus bennetti*				D	LC	Au
Torresian Crow *Corvus orru*	I				LC	Au
Little raven *Corvus mellori*		S			LC	Au
Carrion crow *Corvs corone*	I				LC	Eu
Jackdaw *Corvus monedula*	S				LC	Eu
Rook *Corvus frugilegus*			D		LC	Eu
American crow *Corvus brachyrhynchos*			I		LC	Au
White-necked raven *Corvus albicollis*				D	LC	Afr
Sturnidae						
Wattled starling *Creatophora cinerea*				S	LC	Afr

**Species**	**Res**	**Par**	**Migr**	**Nom**	**Status**	**Region**
Starling *Sturnus vulgaris*		D			LC	Eu
Spotless starling *Sturnus unicolor*	I				LC	Eu
Red-billed starling *Poliopsar sericeus*		S			LC	As
Wattled starling *Creatophora cinerea*				S	LC	Au
Great myna Acridotheres grandis	S				LC	As
Amethyst starling *Cinnyricinclus leucogaster*			D		LC	Afr
Shelley's starling *Lamprotornis shelleyi*			S		LC	Afr
Phloceidae						
scaly-feathered weaver *Sporopipes squamifrons*				S	LC	Afr
Speckle-fronted weaver *Sporopipes frontalis*	S				LC	Afr
Chestnut-crowned Sparrow-weaver *Plocepasser*		S			LC	Afr
Red-headed quelea *Quelea erythrops*			S		LC	Afr
Estrlididae						
Pin-tailed parrotfinch *Erythrura prasina*		S			LC	As
Green-faced parrotfinch *Erythrura viridifacies*				D	V	As
Mount Katanglad parrotfinch *Erythrura coloria*	S				NT	As
Green avadavat *Amandava formosa*	D				V	As
Red avadavat *Amandava amandava*			S		LC	As
Zebra waxbill *Amandava subflava*				S	LC	Afr
Locustfinch *Paludipasser locustella*		S			LC	Afr
Frigillidae						
Ethiopian siskin *Serinus nigriceps*	S				LC	Eu
Cape canary *Serinus canicollis*				S	LC	Afr
Red-fronted serin *Serinus pusillus*			S		LC	As
Syrian serin *Serinus syriacus*		D			V	As
Pine siskin *Carduelis pinus*			S		LC	Am
Andean siskin *Carduelis spinescens*				S	LC	SAm
Olivaceous siskin *Carduelis olivacea*	D				LC	SAm
Eurasian siskin *Carduelis spinus*		S			LC	Eu
black-headed canary *Alario alario*				S	LC	Afr
Blue chaffinch *Fringilla teydea*	I				NT	Eu
Chaffinch *Fringilla coelebs*		S			LC	Eu
Brambling *Fringilla montifringilla*			D		LC	Eu
Emberizidae						
lark-like bunting *Emberiza impetuani*				S	LC	Afr
Godlewski's bunting *Emberiza godlewskii*	S				LC	As
Yellowhammer *Emberiza citrinella*		D			LC	Eu
White-capped bunting *Emberiza stewarti*			S		LC	As
**Trends**						
**Decreasing**	**31**	**18**	**31**	**21**		
**Stable**	**32**	**23**	**15**	**35**		
**Increasing**	**15**	**8**	**6**	**10**		

Taxonomy, movement categories and origin are derived from del Hoyo et al. (1992 - 2002a; vol 1-7) and del Hoyo et al. (2003 – 2011b; vol 8-16). Population trends and status are from IUCN Red List (2015) except for * which are from del Hoyo et al. (1992)

Res: Resident; Par: Partial migrant; Migr: Migrant; Nom: Nomad; Afr: Africa; Am: North America; As: Asia; Au: Australia; Eu: Europe; Sam: South America; NZ: New Zealand; LC: Least concern; NT: Near threatened; E: Endangered; CE: Critically endangered; S: Stable; D: Decreasing; I: Increasing
